# Role of POMC and AgRP neuronal activities on glycaemia in mice

**DOI:** 10.1038/s41598-019-49295-7

**Published:** 2019-09-10

**Authors:** Aykut Göktürk Üner, Onur Keçik, Paula G. F. Quaresma, Thiago M. De Araujo, Hyon Lee, Wenjing Li, Hyun Jeong Kim, Michelle Chung, Christian Bjørbæk, Young-Bum Kim

**Affiliations:** 1 Department of Endocrinology, Division of Endocrinology, Diabetes, and Metabolism, Beth Israel Deaconess Medical Centre and Harvard Medical School, Boston, MA 02215 USA; 2 Department of Physiology, Faculty of Veterinary Medicine, Adnan Menderes University, Efeler, Aydin 09010 Turkey

**Keywords:** Feeding behaviour, Diabetes

## Abstract

Leptin regulates both feeding and glycaemia primarily through its receptors expressed on agouti-related peptide (AgRP) and pro-opiomelanocortin-expressing (POMC) neurons; however, it is unknown whether activity of these neuronal populations mediates the regulation of these processes. To determine this, we injected Cre-dependent designer receptors exclusively activated by designer drugs (DREADD) viruses into the hypothalamus of normoglycaemic and diabetic AgRP-ires-cre and POMC-cre mice to chemogenetically activate or inhibit these neuronal populations. Despite robust changes in food intake, activation or inhibition of AgRP neurons did not affect glycaemia, while activation caused significant (P = 0.014) impairment in insulin sensitivity. Stimulation of AgRP neurons in diabetic mice reversed leptin’s ability to inhibit feeding but did not counter leptin’s ability to lower blood glucose levels. Notably, the inhibition of POMC neurons stimulated feeding while decreasing glucose levels in normoglycaemic mice. The findings suggest that leptin’s effects on feeding by AgRP neurons are mediated by changes in neuronal firing, while the control of glucose balance by these cells is independent of chemogenetic activation or inhibition. The firing-dependent glucose lowering mechanism within POMC neurons is a potential target for the development of novel anti-diabetic medicines.

## Introduction

Over the last century, obesity-related metabolic diseases have increased dramatically worldwide. Known causes for this phenomenon are increasing life expectancy and changes in environmental and behavioural patterns that include a sedentary lifestyle and the excessive intake of calorically rich foods, which can lead to the development of obesity-related metabolic diseases such as diabetes^1^. In fact, the prevalence of diabetes is projected to increase to 300 million by 2025^1^. Despite recent technological advancements and considerable research, diabetes is still a major threat to human health^2^. Even though several drug options are available for diabetes, many patients do not achieve optimal glycaemic targets, and there is no definitive cure for the disease. Thus, effective therapeutic approaches are urgently needed.

Numerous studies have revealed that leptin, leptin-receptors, melanocortin 4 receptors (MC4Rs), glutamate receptor subunit epsilon-2 receptors (GluN2BRs), pro-opiomelanocortin (POMC)-derived peptides, and Agouti-related peptide (AgRP) in the central nervous system (CNS) of rodents are important in the regulation of blood glucose homeostasis^3–6^. Leptin, through signalling via AgRP and POMC neurons, regulates body weight, feeding and glycaemia. For example, it has been shown that re-expression of leptin receptors in the POMC neurons of leptin receptor deficient mice prevents hyperglycaemia, independent of leptin’s anti-obesity effect^4^. In addition, diabetic ob/ob mice with selective deletion of leptin receptors from AgRP neurons failed to exhibit leptin’s glucose-lowering properties, demonstrating a critical role for AgRP neurons in the arcuate nucleus (ARC) in leptin-mediated anti-diabetic action^3^. Our recent work showing that specific deletion of glutamate receptors (GluN2B) from AgRP neurons normalized glucose levels in severely diabetic ob/ob mice further substantiates the importance of these cells^6^. This suggests that glutamate signalling via N-methyl-d-aspartate (NMDA) receptors (GluN2B) in AgRP neurons has a negative impact on glucose control. Combined, these data indicate that AgRP and POMC neurons can exert major control over glucose homeostasis. However, it remains unknown whether AgRP and/or POMC neuronal activity per se is directly involved in glycaemic control as has been shown for feeding control^7–10^.

Designer receptors exclusively activated by designer drugs (DREADDs) are an advanced pharmacogenetic technology that have been broadly used in the field of neuroscience to directly enhance or inhibit the activities of neurons *in vivo* in a non-invasive manner^11^. It involves the modulation of G protein-mediated pathways in specific cell populations through the use of mutated G protein-coupled receptors (GPCRs) that can be chemogenetically activated by a relatively exclusive, inert form of clozapine, clozapine N-oxide (CNO)^12^. Stimulatory hM3Dq and inhibitory hM4Di DREADDs activated by CNO have been used in the central nervous system to study their impact on cognitive function and feeding behaviour^12^. The current study was designed to determine whether modulation of AgRP or POMC neuronal activity by DREADD is sufficient to alter both blood glucose levels and feeding in normal (normoglycaemic) and diabetic ob/ob mice.

## Results

### Activation or inhibition of AgRP neurons by DREADD in normoglycaemic lean mice and diabetic obese mice does not affect blood glucose levels

**Figure 1:**
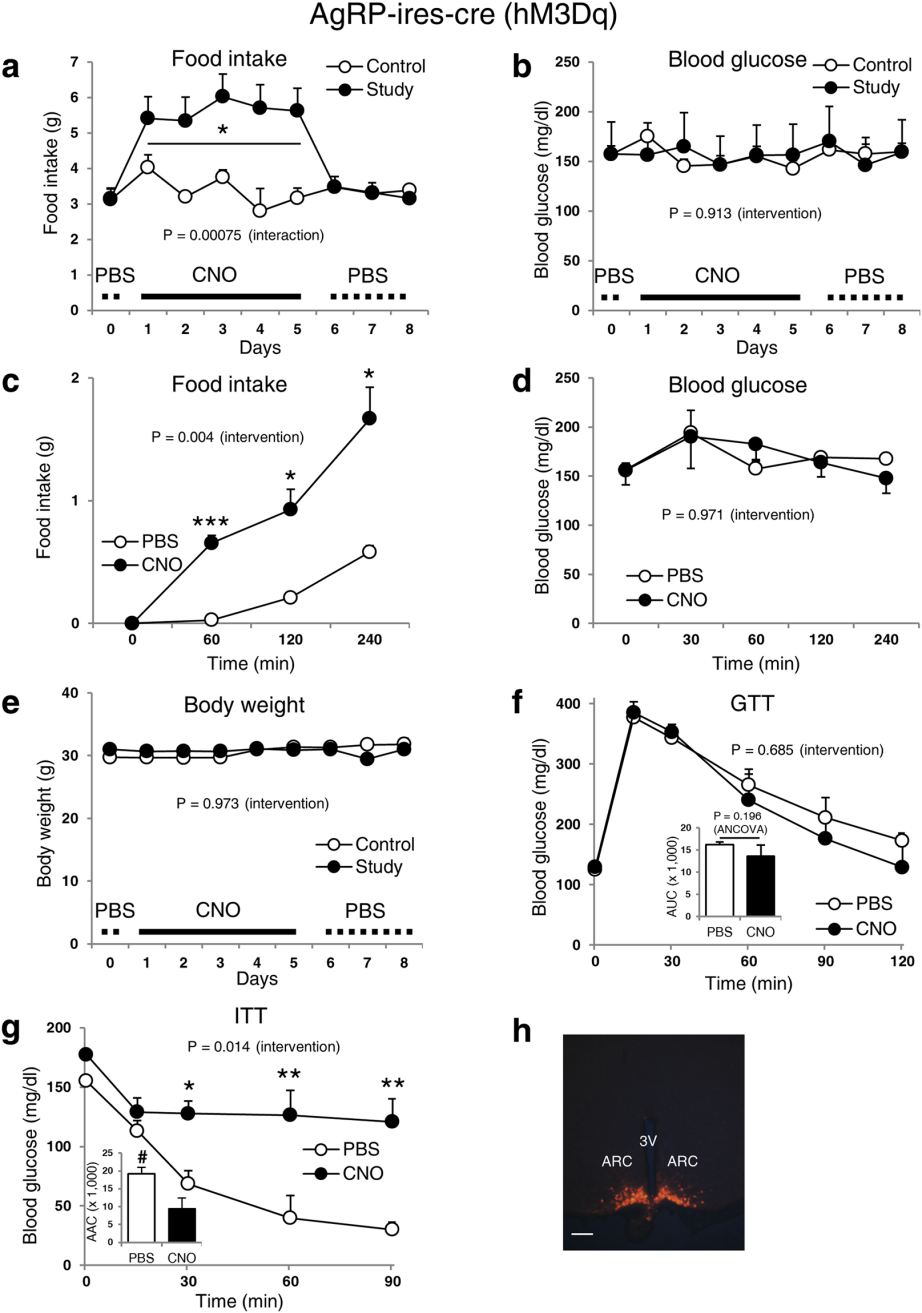
: Stimulation of AgRP neuronal activity in lean normoglycaemic mice affects food intake and insulin sensitivity but not blood glucose levels. Food intake (daily (**a**) and acute (**c**)), blood glucose (daily (**b**) and acute (**d**)), body weight (**e**), GTT (**f**), and ITT (**g**) of AgRP-ires-cre male normoglycaemic mice. (**h**) Representative m-Cherry expression image for the verification of the injection site. Data are shown as means ± s.e.m. (n = 4–6/group). 2-way repeated measures ANOVA was first done to determine the intervention effect or intervention-by-time interactions for all studies except area under the curve (AUC). Then, when intervention differences and/or intervention-by-time interactions were significant by 2-way repeated-measures ANOVA, GLM procedures or 1-way ANCOVA were performed to evaluate the effect of CNO on dependent variables where appropriate. Body weight was used as the covariate while performing 1-way ANCOVA. Logarithmic transformation was done for daily food intake values to normalize variance. AUC or AAC was evaluated with 1-way ANCOVA. CNO (1 mg/kg) was injected every 8 hours daily. AAV8-DIO-hM3Dq-mCherry (activator) was injected into the study and control mice that received CNO and PBS, respectively for 5 days. All mice then received PBS for an additional 3 days. CNO: Clozapine N-oxide. GTT: Glucose tolerance test. ITT: Insulin tolerance test. 3V: Third ventricle. ARC: Arcuate nucleus. The image in (**h**) was captured at 20× magnification (cropped upper side of slide). Scale bar, 100 μm. *P ≤ 0.05, ***P ≤ 0.001, ^#^P ≤ 0.05.

To determine whether activation or inhibition of AgRP neurons can influence blood glucose levels, we first enhanced the AgRP neuronal activity of normoglycaemic AgRP-ires-cre mice by stereotactically injecting AAV8-DIO-hM3Dq-mCherry into the ARC and activated the transduced cells with CNO. Consistent with previous findings^9,10^, activation of AgRP neurons caused a significant (P = 0.00075 and P = 0.004 for daily and acute food intake, respectively) increase in daily and acute food intake (Fig. 1a,c). However, activation of these cells did not alter blood glucose levels (Fig. 1b,d), body weight (Fig. 1e), or glucose tolerance (Fig. 1f). Notably, enhancement of AgRP neuronal activity did result in a significant (P = 0.014) impairment of insulin sensitivity (Fig. 1g). Of note, CNO alone (without DREADD) did not affect body weight, food intake, blood glucose, or GTT in non-cre expressing mice (Supplementary Fig. S1). In addition, the DREADD viruses per se (off-target infection, with or without CNO) did not affect any of the same metabolic parameters (Supplementary Figs S2, S3, and S4).

**Figure 2:**
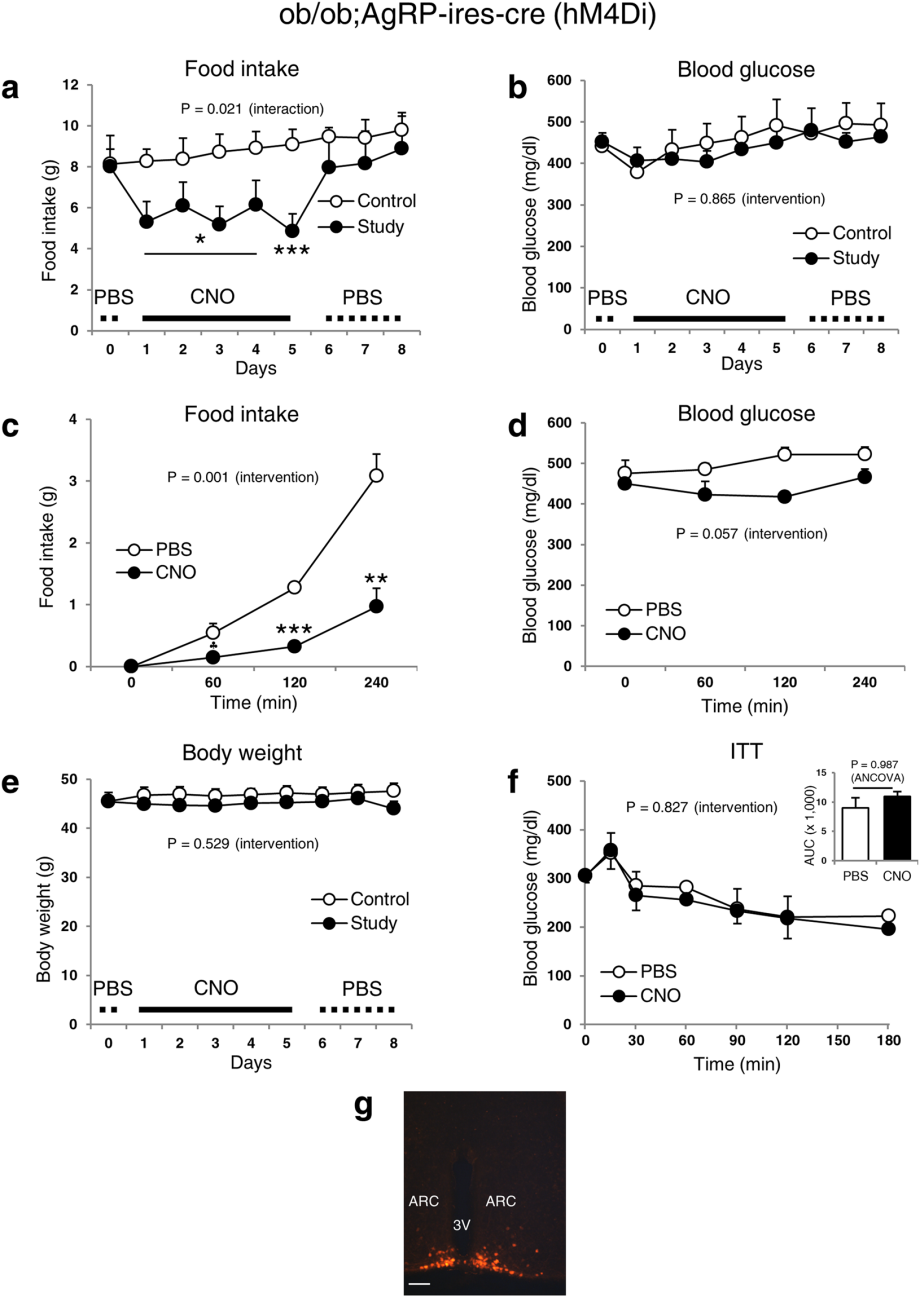
Inhibition of AgRP neuronal activity in obese diabetic mice affects food intake but not blood glucose. Food intake (daily (**a**) and acute (**C**)), blood glucose (daily (**b**) and acute (**d**)), body weight (**e**) and ITT (**f**) of diabetic ob/ob; AgRP-ires-cre male mice. (**g**) Representative m-Cherry expression image for the verification of the injection site. Data are shown as means ± s.e.m. (n = 3–5/group). Logarithmic transformation was done for daily food intake values to normalize variance. Statistics were performed as described in Fig. 1. CNO (1 mg/kg) was injected 3 times a day. AAV8-DIO-hM4Di-mCherry (inhibitory) was injected into the study and control mice that received CNO and PBS, respectively for 5 days. All mice then received PBS for an additional 3 days. Abbreviations are the same as described in Fig. 1. The image in (**g**) was captured at 20× magnification. Scale bar, 100 μm. *P ≤ 0.05, **P ≤ 0.01, ***P ≤ 0.001.

In diabetic ob/ob;AgRP-ires-cre mice injected with AAV8-DIO-hM4Di-mCherry in the ARC, CNO administration markedly decreased daily (by ~46% after 5 days, Fig. 2a) and acute (by ~69% after 4-h, Fig. 2c) food intake without influencing glucose levels (Fig. 2b). The trend towards lower glucose levels at 1 hour after treatment was likely due to a reduction in food intake (Fig. 2d). Serum insulin was also normal in control and CNO-injected AgRP-ires-cre mice (Supplementary Fig. S5). Furthermore, body weight and insulin sensitivity were unaffected during CNO administration (Fig. 2e,f). Together, these data show that, although modulation of hypothalamic AgRP neuronal activity can control food intake, it is insufficient to change blood glucose levels in normal and diabetic mice.

### Activation of AgRP neurons by DREADD is sufficient to attenuate leptin’s effects on food intake but not glycaemia in ob/ob mice

**Figure 3:**
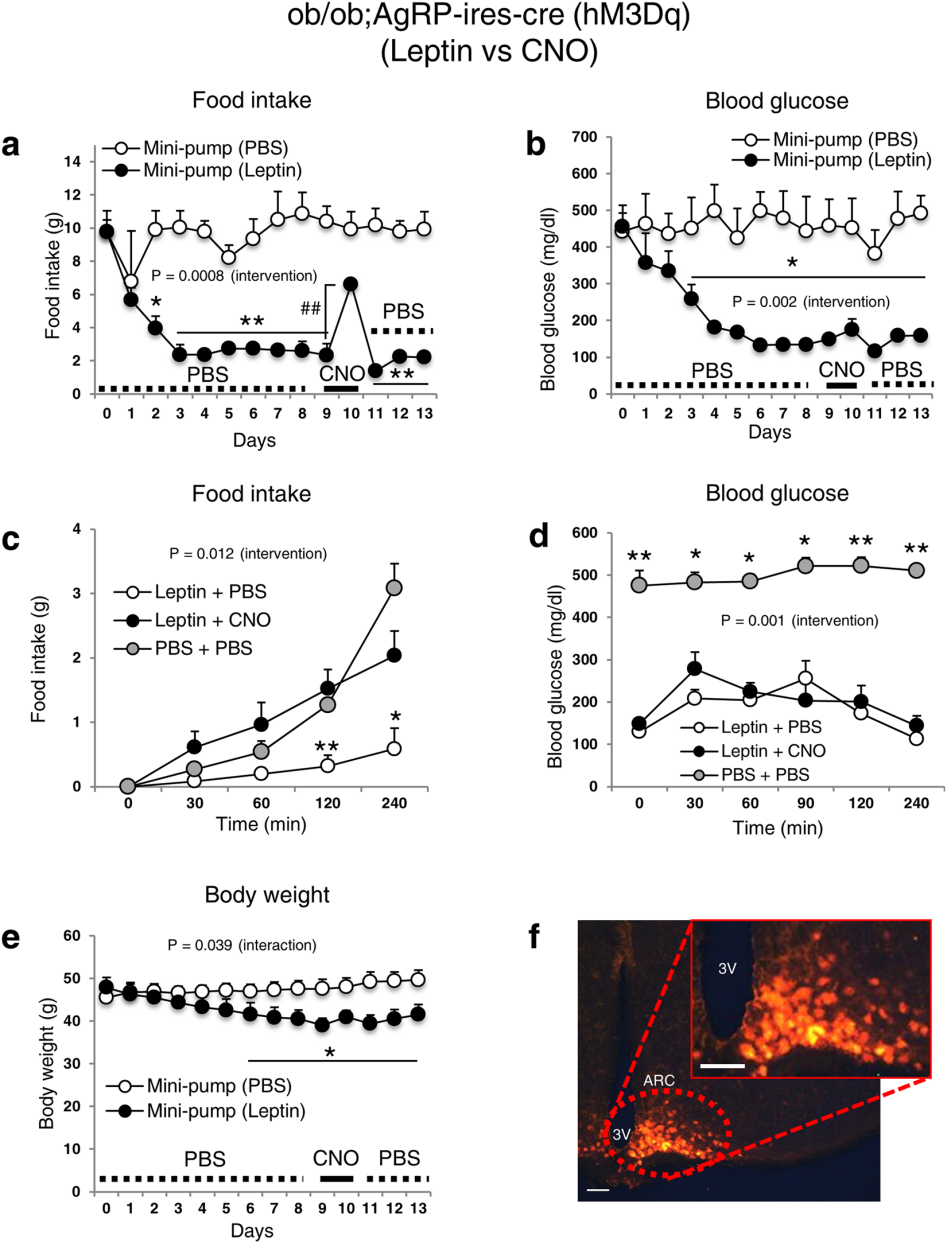
Stimulation of AgRP neuronal activity in ob/ob mice is sufficient to attenuate the effect of leptin on food intake, but not on blood glucose. Food intake (daily (**a**) and acute (**c**)), blood glucose (daily (**b**) and acute (**d**)), body weight (**e**) of diabetic ob/ob; AgRP-ires-cre male mice that were implanted with an osmotic mini-pump loaded with leptin (500 ng/h for 13 days). (**f**) Representative m-Cherry expression image for the verification of the injection site. Data are shown as means ± s.e.m. (n = 4/group). Statistics were performed as described in Fig. 1. P -value (interaction) in (**e**) was obtained by Greenhouse-Geisser correction. CNO administration at day 9 led to an increase in body weight of mice infused with leptin at day 10 (P = 0.007, paired t-test). AAV8-DIO-hM3Dq-mCherry (activator) was injected into the leptin- or PBS-injected ob/ob mice. CNO (1 mg/kg) or PBS was injected 5 times a day at day 9 or before the acute experiments ((**c**) and (**d**)). Abbreviations are the same as described in Fig. 1. The image in (**f**) was captured at 20× magnification accompanied by another image at higher magnification. Scale bars, 60 μm. *P ≤ 0.05 [different from other group(s)], **P ≤ 0.01 [different from other group(s)]. P ≤ 0.01 between day 9 and day 10.

Since AgRP neurons are required for leptin’s glucose-lowering properties^3^ and are known to be inhibited by leptin^13,14^, we further examined whether the activation of AgRP neurons by the excitatory DREADD virus AAV8-DIO-hM3Dq-mCherry could antagonize the leptin-induced normalization of hyperglycaemia in diabetic mice (ob/ob;AgRP-ires-cre). Animals were fitted with osmotic leptin- or PBS-filled mini-pumps for 8 days. After 3–4 days, food intake and blood glucose levels normalized (Fig. 3a,b). On day 9, we injected CNO five times within a 24 h period to maximally stimulate AgRP neurons. CNO-treatment increased food intake by 284% (P = 0.003) (Fig. 3a), but blood glucose levels were unaffected (Fig. 3b). Similarly, acute food intake increased remarkably by CNO administration (Fig. 3c) while blood glucose remained unchanged (Fig. 3d). Body weight data are shown in Fig. 3e. Collectively, these results suggest that the modulation of DREADD-induced AgRP neuronal activity per se is not sufficient to influence blood glucose homeostasis.

### Inhibition of POMC neurons by DREADD reduces blood glucose levels in normoglycaemic mice

**Figure 4:**
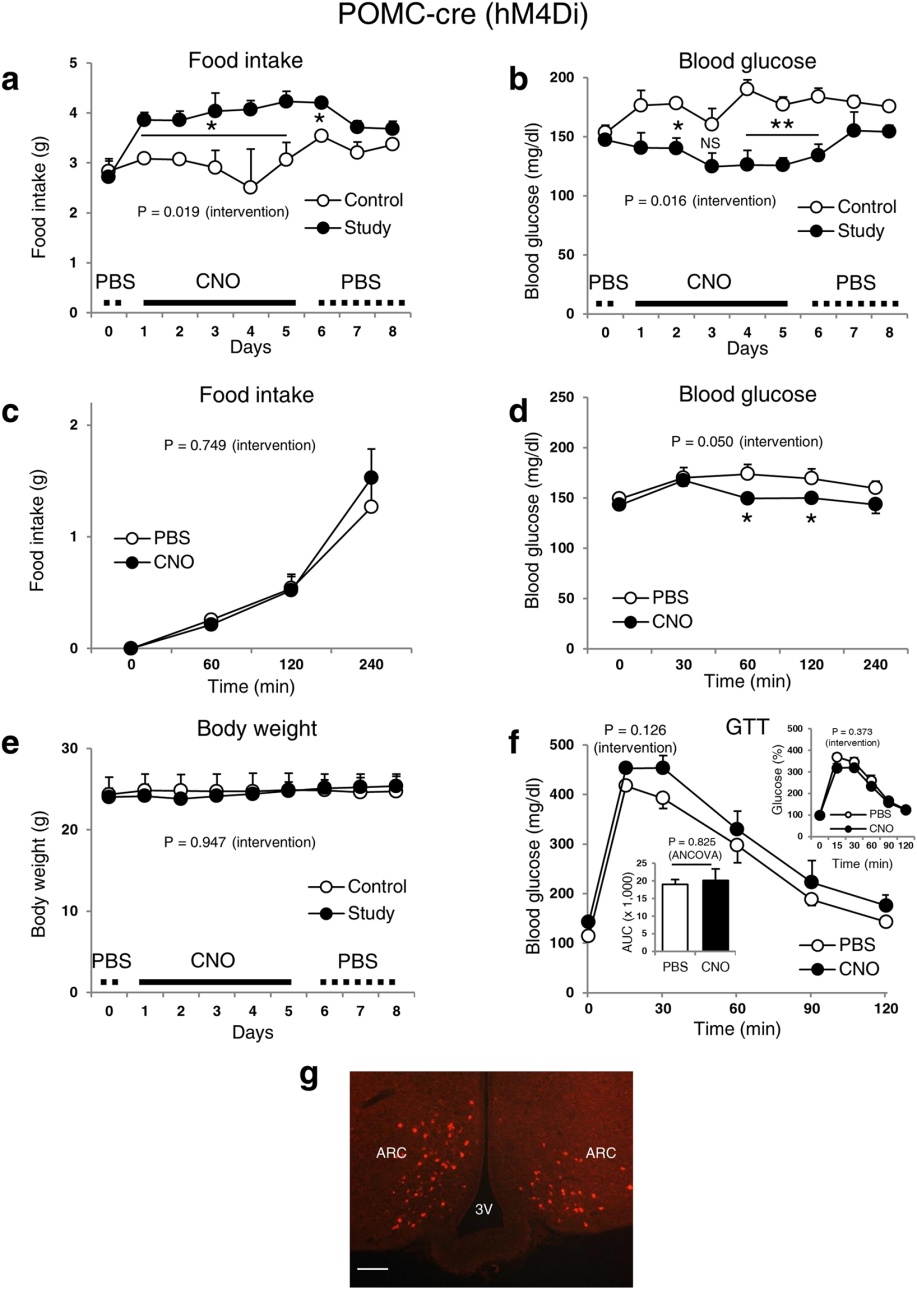
Inhibition of POMC neuronal activity in lean normoglycaemic mice increases food intake, but decreases blood glucose. Food intake (daily (**a**) and acute (**c**)), blood glucose (daily (**b**) and acute (**d**)), body weight (**e**) and GTT (**f**) of POMC-cre male normoglycaemic mice. (**g**) Representative m-Cherry expression image for the verification of the injection site. Data are shown as means ± s.e.m. (n = 4–5/group). Statistics were performed as described in Fig. 1. CNO (2.5 mg/kg) was injected every 6 hours. AAV8-DIO-hM4Di-mCherry (inhibitory) was injected into the study and control mice that received CNO and PBS, respectively for 5 days. All mice then received PBS for an additional 3 days. Abbreviations are the same as described in Fig. 1. The image in (g) was captured at 20× magnification. Scale bar, 100 μm. NS: Not significant. *P ≤ 0.05, **P ≤ 0.01.

To determine whether changes in POMC neuronal activity can alter blood glucose levels, we inhibited POMC neurons by injecting AAV8-DIO-hM4Di-mCherry into the ARC of normoglycaemic POMC-cre mice and monitored food intake and blood glucose levels before and after CNO treatment. In agreement with previous findings^8^, inhibition of POMC neurons by DREADD increased daily food intake (Fig. 4a); however, we did not observe any changes in acute food intake (Fig. 4c). Interestingly, we also found that CNO significantly reduced blood glucose levels (both acutely and daily) in normoglycaemic POMC-cre mice (Fig. 4b,d). Body weight and GTT showed no change (Fig. 4e,f).

**Figure 5:**
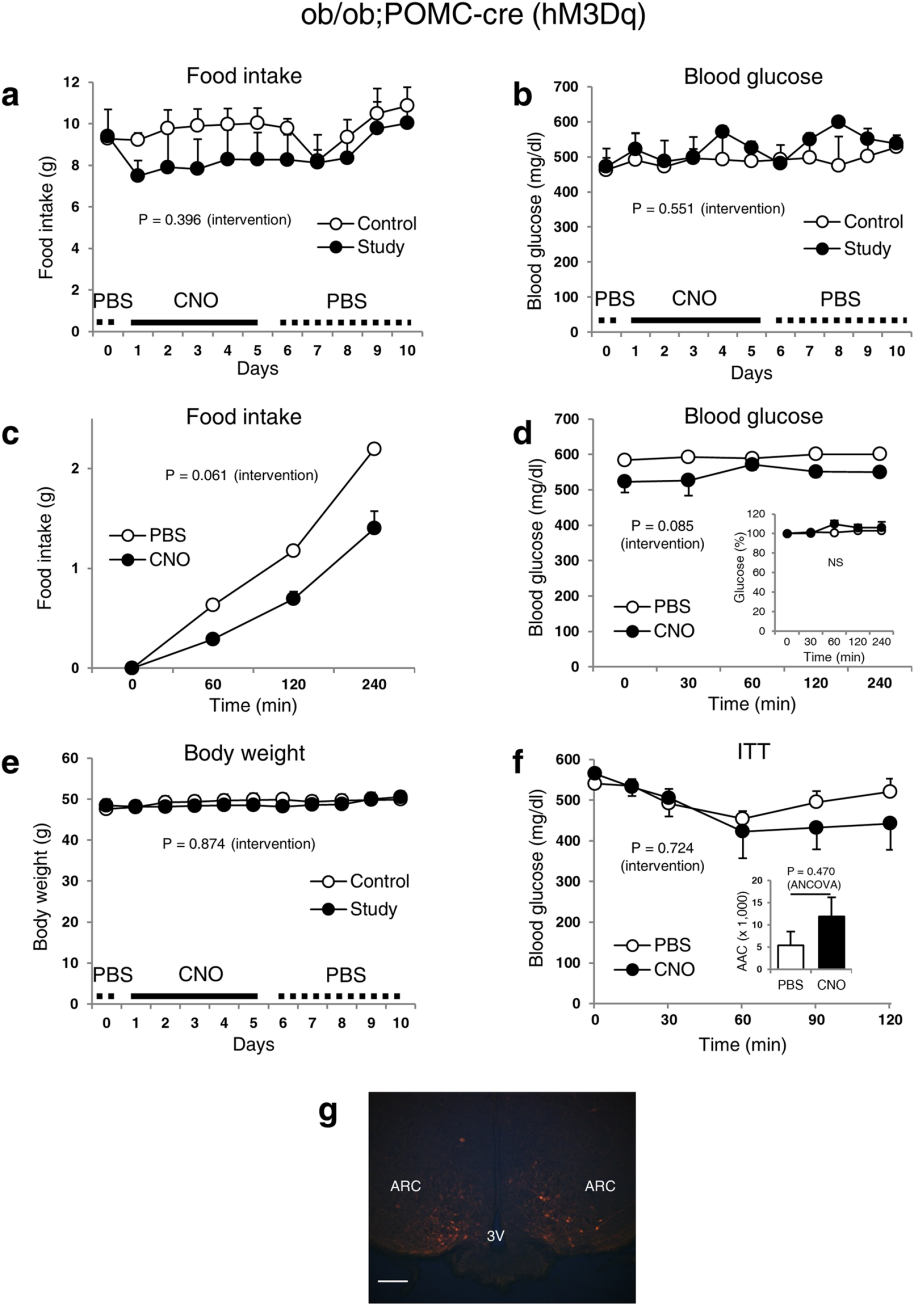
Stimulation of POMC neurons in diabetic ob/ob mice does not influence blood glucose levels. Food intake (daily (**a**) and acute (**c**)), blood glucose (daily (**b**) and acute (**d**)), body weight (**e**) and ITT (**f**) of diabetic ob/ob; POMC-cre male mice. (**g**) Representative m-Cherry expression image for the verification of the injection site. Data are shown as means ± s.e.m. (n = 3–5/group). Statistics were performed as described in Fig. 1. Area above the curve (AAC) was evaluated with 1-way ANCOVA. AAV8-DIO-hM3Dq-mCherry (activator) was injected into the study and control mice that received CNO and PBS, respectively for 5 days. All mice then received PBS for an additional 5 days. CNO (2.5 mg/kg) was injected every 6 hours. Abbreviations are the same as described in Fig. 1. The image in (**g**) was captured at 20× magnification. Scale bar, 120 μm. NS: Not significant.

We then injected of AAV8-DIO-hM3Dq-mCherry into the ARC of obese, diabetic ob/ob;POMC-cre mice to determine whether stimulation of POMC neurons affects blood glucose levels. An acute single injection of CNO (Fig. 5c) or continuous CNO administration for 5 days (Fig. 5a) showed a trend toward decreased food intake although this did not reach statistical significance (P = 0.061, acute experiment). Blood glucose levels (Fig. 5b,d), body weight (Fig. 5e), and ITT (Fig. 5f) were unaffected by CNO administration. These data suggest that the modulation of POMC neuronal activity using DREADD can alter glycaemic levels in normoglycaemic mice, but not mice in an obese, diabetic state. Because leptin is known to increase the firing rate of POMC neurons as well as lower blood glucose levels via leptin receptor-mediated pathways in the ARC^4,15^, the current finding showing that the inhibition of POMC neurons by DREADD reduces circulating glucose, is surprising.

### DREADD-dependent inhibition of POMC neurons lowers glucose independently of food intake

**Figure 6:**
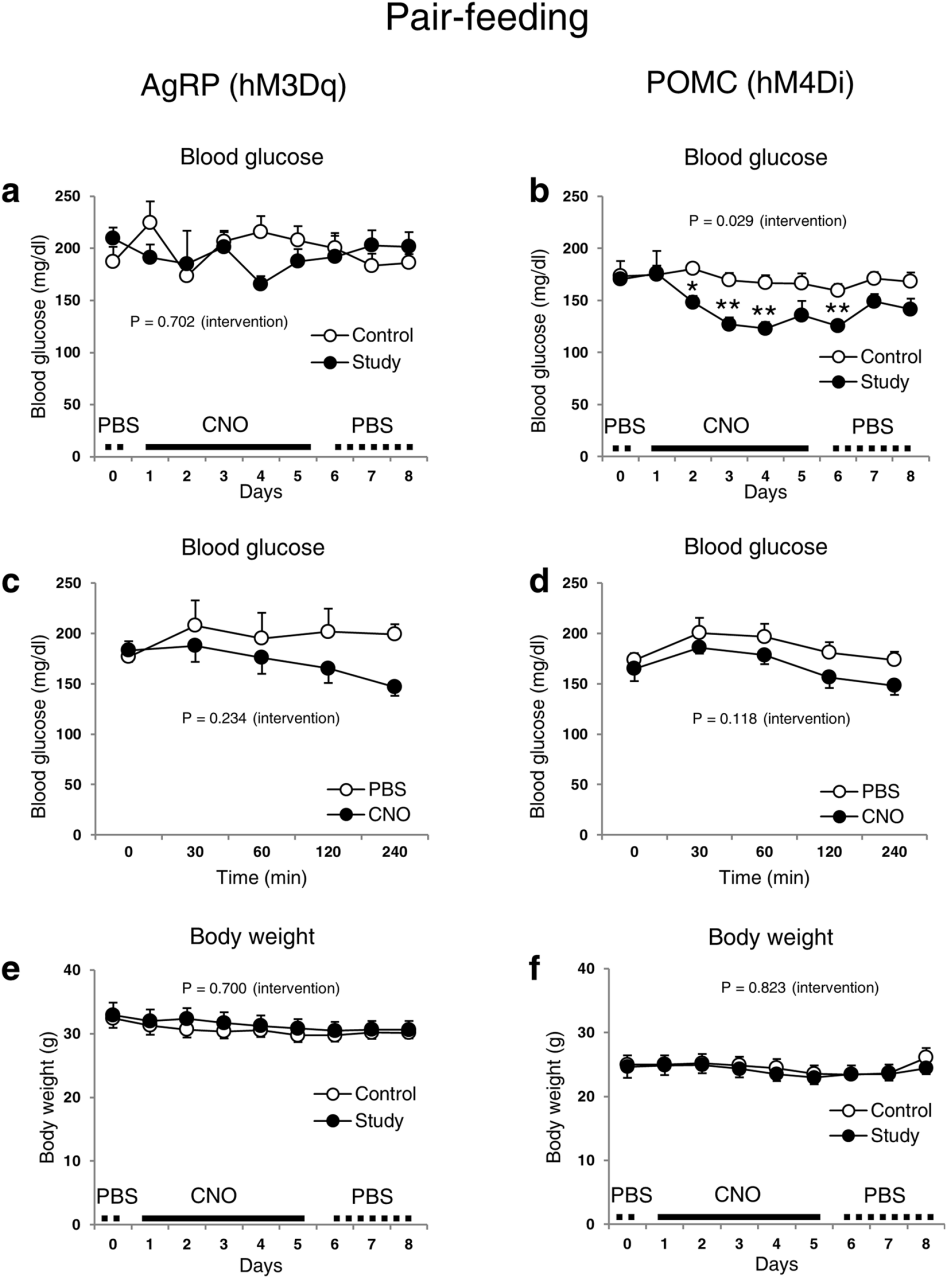
Regulation of AgRP and POMC neurons by DREADD: Pair-feeding studies. Daily (**a**) and acute (**c**) blood glucose and body weight (**e**) of normoglycaemic AgRP-ires-cre male mice. Daily (**b**) and acute (**d**) blood glucose and body weight (**f**) of normoglycaemic POMC-cre male mice. Data are shown as means ± s.e.m. (n = 4–10/group). AAV8-DIO-hM3Dq-mCherry (activator) and AAV8-DIO-hM4Di-mCherry (inhibitory) were injected into the arcuate nucleus of AgRP-ires-cre and POMC-cre mice, respectively. CNO (1–2.5 mg/kg) or PBS was injected every 6 or 8 hours for 5 days. All mice then received PBS for an additional 3 days. All experiments were done under pair-feeding conditions. Abbreviations are the same as described in Fig. 1. Statistics were performed as described in Fig. 1.

Because food intake was altered by the administration of CNO in virus injected AgRP-ires-cre and POMC-cre mice, we performed pair-feeding studies to determine whether the observed changes in blood glucose levels were due to changes in caloric intake. Under pair-feeding conditions, blood glucose levels were not different between PBS-injected and CNO-injected AgRP-ires-cre mice expressing AAV8-DIO-hM3Dq-mCherry (Fig. 6a,c), which is consistent with earlier data (Fig. 1). Importantly, POMC-cre mice injected with AAV8-DIO-hM4Di-mCherry maintained lower glucose levels following CNO treatment in pair-fed mice (Fig. 6b). Acute blood glucose levels also tended to decrease in response to CNO in these mice (Fig. 6d). These data show that inhibition of POMC neuronal activity lowers blood glucose levels independently of energy intake.

## Discussion

Leptin administration to obese, diabetic mice and insulin-deficient animals can entirely correct blood glucose levels^3,16^. This anti-diabetic effect is mediated, at least in part, by POMC and AgRP neurons^3^. Leptin therapy also greatly improves glycaemia in humans suffering from lipodystrophy^5,17^. Therefore, hypothalamic AgRP and POMC neurons may play important roles in the regulation of human glucose metabolism. Yet, the specific role of neuronal activity per se in these populations remains undetermined. In this study, we discovered that silencing POMC neuronal activity using DREADD technology in mice leads to a marked reduction in blood glucose levels, independent of food intake. In contrast, neither activation nor inhibition of AgRP neurons by DREADD affects blood glucose balance.

Studies have demonstrated that changes in action potential firing rates of AgRP neurons after chemogenetic (DREADD) and optogenetic modulation can rapidly alter energy intake^7,9^. Our results show that, despite a robust impact on food intake, DREADD-mediated suppression or stimulation of AgRP neuronal activity is not sufficient to affect blood glucose levels, thus pointing to divergent cellular mechanisms that regulate feeding and glucose balance by leptin. In support of our findings, a recent study similarly reported that DREADD-mediated activation of AgRP neurons had no effect on blood glucose levels, although systemic insulin sensitivity was decreased^10^. We also confirm this worsening in insulin sensitivity (Fig. 1g) without affecting systemic glucose tolerance. In contrast, a separate study showed that activation of inhibitory DREADDs in AgRP neurons can modestly improve glycaemia in STZ-induced diabetic mice^18^. The reason for the discrepancy is unclear, but it may include the use of different diabetic models or a marked reduction in food intake that could drive a secondary lowering of blood glucose levels.

Leptin’s action on orexigenic AgRP-expressing neurons in the ARC has been well documented^3^. Leptin receptor signalling affects numerous major neuronal processes such as; neuronal firing^13,19^, axonal/neurite outgrowth^20^, dendritic branching^21^, neurotransmitter/neuropeptide release^22^, gene transcription^23^, spine formation^24^, and synaptogenesis^25^. There are differences between DREADD-mediated and leptin receptor pathways, and if fully understood, could provide important information on the nature of these critical processes. For example, the leptin receptor canonically signals via the “Janus kinase 2 - signal transducer and activator of transcription 3” (JAK2-STAT3) pathway^26^, which is not typically engaged by the GPCR class to which the DREADDs belong. Instead, they normally signal via the cyclic AMP/protein kinase A (cAMP/PKA; Gi) or phospholipase C/protein kinase C/calcium (PLC/PKC/Ca^2+^; Gq) pathways^11,12,27,28^. We have recently reported that deletion of the GluN2B subunit of NMDARs from AgRP neurons normalizes hyperglycaemia in severely diabetic Lep^ob/ob^ mice^6^. This effect is likely caused by decreased glutamate action in AgRP neurons as well as changes in synaptogenesis^29^. DREADDs, on the other hand, primarily affect action potential firing, not slower processes such as synaptogenesis. Consistent with this conclusion, we found that the effects of DREADD on feeding were rapid (<60 min) whereas the effects of leptin on glucose were much slower (>1 day). Leptin receptor-mediated and NMDAR-mediated signalling could both trigger similar AgRP neuronal processes that influence downstream circuits, which modulate peripheral glucose regulatory systems^30,31^, while the GPCR-mediated DREADD signalling in AgRP neurons may not^32^. On the other hand, there could be overlaps in the signalling between leptin receptors and DREADDs, which may include mitogen-activated protein kinase (MAPK), Rho-associated protein kinase (ROCK) and phosphoinositide 3-kinase (PI3K)^26,30,33–38^. Of note, the GPCRs that are used in DREADD technology are modified (artificial) receptors and their roles on individual intracellular signalling pathways *in vivo* are not completely known. Further studies are needed to verify pathways that overlap with or diverge from leptin signalling, since such information could be exploited to identify potential targets that are crucial in leptin’s anti-diabetic action.

We found that the DREADD-induced inhibition of POMC neurons in the ARC of lean, normoglycaemic mice lowered systemic blood glucose levels. This surprising because leptin is known to activate POMC neurons, induce an anorexigenic response and prevent hyperglycemia^4,39^. Our finding leads to the questions of which *in vivo* mechanisms are involved and what physiological roles do they play. One interesting possibility that might explain our unexpected result is the reported existence of several POMC sub-populations in the hypothalamus^40^. Lam *et al*. showed that insulin and leptin act dissociatively on distinct groups of POMC neurons, with leptin activating one population and insulin inhibiting a separate subpopulation of POMC cells. In our study, we used POMC-cre mice to express DREADD-receptors, thus any of the POMC-expressing neuronal subpopulations that were infected could have effectively been manipulated chemogenetically. It is therefore possible that the lowering of blood glucose levels observed by DREADD inhibition reflects the action of the POMC-subpopulation that is normally inhibited by insulin. Future studies using mice that express DREADD-hM4Di exclusively in insulin-responsive POMC neurons would need to be performed to verify this hypothesis. To do this, Pomc^NEO^ mice expressing IR-Cre (IR^Cre/+^; Pomc^NEO/NEO^) could be generated^40,41^, or a more specific technology called INTRSECT (INTronic Recombinase Sites Enabling Combonatorial Targeting), in which Cre and Flp recombinases are combined and inserted with the insulin-receptor coding sequence site in POMC-ires-cre mice^42^, could be used to studied the effects of this specific subpopulation of neurons on feeding and glycaemic control. In addition, there might also be similar or shared intracellular processes between inhibitory DREADDs and insulin. Indeed, both DREADDs and insulin target potassium channels in neurons, although insulin and inhibitory DREADDs use different potassium channels as well as different intracellular signalling cascades^11,12,27,28,43^. The physiological roles of insulin and leptin’s gluco-regulatory actions on select populations of POMC neurons and the intracellular pathways that govern them are interesting areas of future studies. Of note, acute inhibition of POMC neuronal activity did not lead to an increase in food intake. This may be due to the dichotomous nature of the control of food consumption by POMC neurons. It is known that two different populations of POMC neurons located in the ARC and brain stem affect feeding behaviour by mediating long-term processes and short-term processes, respectively^44^.

The current study did not directly measure neuronal activity of AgRP or POMC neurons after infection with the DREADD virus, which is a limitation of this investigation. However it has been well-established that changes in neuronal activity occur with the expression of specific DREADD viruses, where the Gq-coupled stimulatory DREAD hM3Dq enhances neuronal firing, and the Gi/o-coupled inhibitor DREAD hM4Di inhibits action potential firing after pharmacological CNO administration^9^. In this study, transduction of genetic DREADD genetic material by AAV infection was confirmed using immunohistochemistry. We found that 1,398 ± 264 neurons per mouse brain (mean ± s.e.m.; range 448–2408; n = 6 mice) were mCherry positive in AgRP-ires-cre mice (Supplementary Fig. S6), which was consistent with a report showing that ~700 AgRP neurons were sufficient to stimulate feeding by photostimulation^7^. We also found that 1,182 ± 245 neurons per mouse brain (mean ± s.e.m.; range 496–2520; n = 7 mice) were positive for mCherry in POMC-cre mice (Supplementary Fig. S6). After infection with DREADD hM3Dq or hM4Di, food intake results were used as an indication of CNO-induced AgRP or POMC neuronal activity, which were similar to findings reported earlier^7–9^. These data strongly suggest that DREADD-mediated neuronal activity is functional under our experimental conditions. Another limitation of this study was the use of diabetic ob/ob mice to show the possible anti-diabetic effects of chemogenetic modulation on AgRP and POMC neurons. Although ob/ob obese mice are a useful and well-studied model of obesity and diabetes, monogenetic mouse models do not closely mimic the pathomechanisms^45–49^ that are believed to be involved in the development of human diabetes. Therefore, future studies including mice fed with high-fat diet or injected with streptozotocin could yield interesting results with regards to human disease.

**Figure 7:**
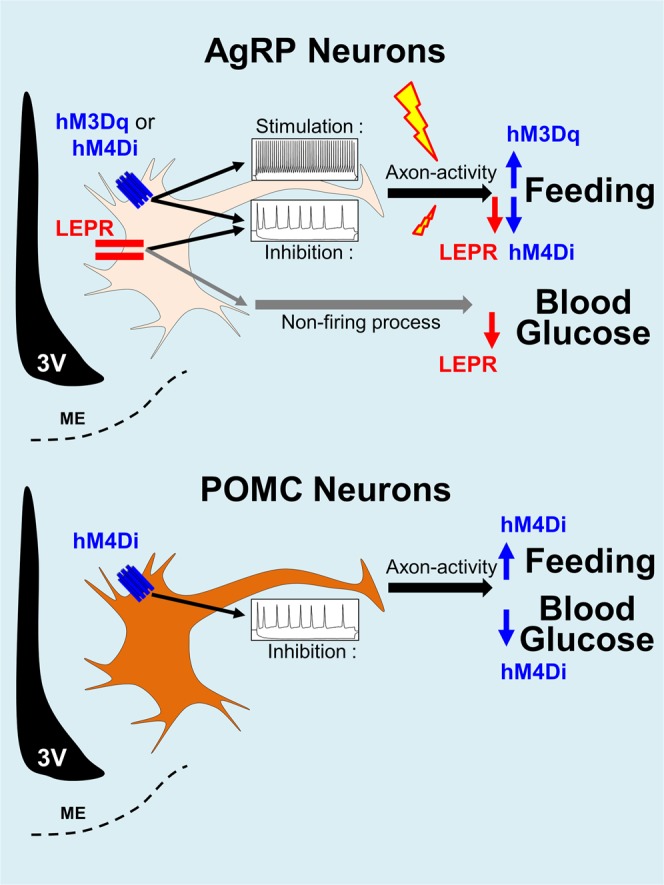
Schematic representation of mechanisms within AgRP and POMC neurons that control feeding and blood glucose. 3 V: Third ventricle. ME: Median eminence. LEPR: Leptin receptor.

In summary, the present research demonstrates that; (i) DREADD-mediated selective inhibition of AgRP neurons or DREADD-mediated activation of POMC neurons in ob/ob mice does not affect glycaemia, despite clear and expected effects on feeding; (ii) activation of AgRP neurons is neither sufficient nor required for leptin’s glucose-lowering affect; (iii) in contrast to the reduction of blood glucose levels following the stimulation of POMC neurons by leptin, silencing of POMC neurons in the hypothalamus by DREADD is sufficient to lower glucose levels in normoglycaemic mice; and (iv) in contrast to the control of feeding, the neuronal mechanisms required for leptin’s beneficial effects on glucose homeostasis via AgRP and POMC neurons are independent of chemogenetic activation or inhibition. These data demonstrate that the glucose-lowering action of leptin may not depend on the modulation of AgRP action potential firing, while the inhibition of POMC neuronal firing appears to lower blood glucose levels. This is clearly divergent from evidence that changes in the firing rates of these neuronal populations regulate feeding (Fig. 7). Targeting firing-dependent mechanism in POMC neurons may lead to novel therapies for glycaemic control.

## Methods

### Animal care

All aspects of animal care and experiments were conducted according to the National Institutes of Health “Guide for the Care and Use of Laboratory Animals” (NIH Publication No. 85-23, revised 1996) and were approved by the Institutional Animal Care and Use Committee of Beth Israel Deaconess Medical Center. Mice were housed at 22–24 °C with a 12 h light/12 h dark cycle and ad libitum access to standard pelleted chow and water.

### Generation of experimental animals

The generation of AgRP-ires-cre and POMC-cre mice was described previously^50–52^. To generate ob/ob; AgRP-ires-cre or ob/ob; POMC-cre mice, we first obtained ob/+; AgRP-ires-cre (or ob/+; POMC-cre) breeders by mating AgRP-ires-cre (or POMC-cre) and ob/+mice (Lep^ob/+^, Jackson Laboratory. Stock no: 000632 [B6.Cg-Lep^ob/J^]). After collecting ob/+; AgRP-ires-cre (or ob/+; POMC-cre) breeders, we crossed them with each other (ob/+; AgRP-ires-cre X ob/+; AgRP-ires-cre or ob/+; POMC-cre X ob/+; POMC-cre) to generate ob/ob; AgRP-ires-cre and ob/ob; POMC-cre mice. Age-matched male littermates were used in the experiment. Genotyping for AgRP-ires-cre, POMC-cre, and ob was done as described previously^50,51,53^.

### Adeno-associated virus injection and mCherry positive cell counting

Cre-dependent AAV8-DIO-hM3Dq-mCherry (activator) or AAV8-DIO-hM4Di-mCherry (inhibitory) was purchased from the University of North Carolina Vector Core (Chapel Hill, NC). Fifty to 100 nl of AAV were bilaterally injected into the ARC of the experimental mice. These mice were anesthetized using 40 mg/kg ketamine and 10 mg/kg xylazine. Stereotaxic coordinates for injection were AP: −1.50 mm, DV: −5.80 mm, and ML: ±0.20 mm from the bregma. Stereotaxic and surgical procedures were carried out as described previously^54^. Briefly, mice were anesthetized and were then placed into a stereotaxic instrument. A small incision was made to expose the skull and the bregma, after which AAV8-DIO-hM3Dq-mCherry or AAV8-DIO-hM4Di-mCherry was pulled into a glass pipette. To inject the virus, a small hole was drilled, after the stereotaxic coordinates for the ARC were determined. The virus was delivered by an air pressure system (AstroNova, Inc., RI [formerly Astro-Med, Inc.]). The delivery rate was ~20–40 nL/min. After the injection, before removing the pipette, we waited for 5 minutes to allow the virus to diffuse into the area. The incision was closed using medical skin glue. Meloxicam (5 mg/kg, subcutaneous) was injected for postoperative care for 2 days. Verification of the injection site was confirmed by the location of m-Cherry expression in coronal brain sections under a fluorescent microscope at the end of studies (see Figs 1–5 for representative examples)^54^. Only mice with verified m-Cherry expression were used for analysis. Mice with virus injections that missed the target area, partial hits, or m-Cherry expression that extended beyond the targeted area were excluded from the study. To quantify mCherry positive (infected) AgRP or POMC neurons, one of four coronal brain series (8–10 sections/series) was mounted onto microscope slides (VWR Scientific, Radnor, PA). The images were taken using a fluorescent microscope and recorded. The number of infected cells was then counted manually in all sections from the recorded images. To estimate the total infected cell number, the counted cells were multiplied by four since there were four series^55^. The counting of AgRP or POMC cells infected by the DREADD virus and injections that missed the target site are shown in Supplementary Fig. S6.

### Food intake, blood glucose, body weight, insulin measurements and pair-feeding

For activation or inhibition of AgRP and POMC neurons, CNO (1–2.5 mg/kg) was administered intraperitoneally in AAV-injected normoglycaemic mice and diabetic obese mice expressing Cre recombinase. For acute studies, food intake, blood glucose, and body weight were measured at 0, 1, 2, and 4 hrs after PBS/CNO injection between 5 pm and 9 pm (or 9 am and 2 pm). For daily studies, CNO was injected 3–5 times a day for 5 days, and food intake, blood glucose, and body weight were measured between 10:00 am and 11:00 am for 10 days. Blood glucose levels were measured using the OneTouch Ultra glucometer (LifeScan Inc., Milpitas, CA). Serum insulin was measured by ELISA (Crystal Chem, Elk Grove Village, IL). For pair-feeding studies, the same amount of food provided to the control mice was given to the CNO-injected mice^56^.

### Glucose (GTT) and insulin (ITT) tolerance tests

**Figure 8:**
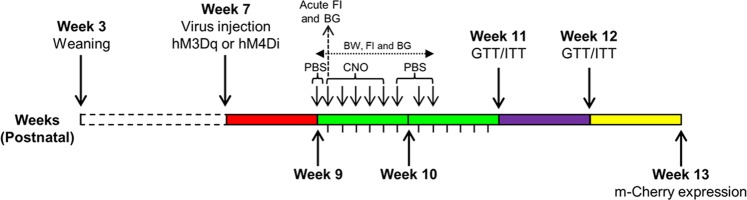
Timeline of experimental procedures. PBS or CNO (1–2.5 mg/kg) was injected 3–5 times per day depending on the experiment. Measurement of acute food intake and blood glucose was done on the first day of the CNO (week 9, day 64) injection period. For GTT and ITT measurements, PBS or CNO was injected 30–60 min before the experiment. The timeline does not include the leptin infusion study. Osmotic mini-pumps filled with leptin were implanted at 62 days of age for 2 weeks. During leptin infusion, CNO was injected at day 9. PBS was also injected daily before and after CNO injection. ARC: Arcuate nucleus. FI: Food intake. BG: Blood glucose. CNO: Clozapine N-oxide. GTT: Glucose tolerance test. ITT: Insulin tolerance test.

GTT and ITT were performed 30 min or 60 min after PBS or CNO injections (see Fig. 8). Food was removed 15 h and 5 h before GTT and ITT tests, respectively. Blood glucose levels were measured at baseline, 15, 30, 60, 90, and 120 min (and 180 min for ob/ob; AgRP-ires-cre mice) after intraperitoneal injection of glucose (2 g/kg) or insulin (1–2.5 U/kg)^6^.

### Leptin administration

Leptin (500 ng/h) was administered via osmotic mini-pumps for 13 days. The Alzet pump (model 2002) was implanted subcutaneously after intraperitoneal injection of ketamine-xylazine^3,4,6^. Briefly, after shaving, a small incision was made in the interscapular area. The incision was then enlarged with a scissor or haemostat to make a pocket for the pump. The pump was then implanted and the incision was closed with a wound clip. Body weight, food intake, and blood glucose levels were measured every morning between 10:00 am and 11:00 am for 13 days. CNO or PBS was injected at day 9 after surgery.

### Statistical analysis

Power analysis was done to estimate sample size using the GPower 3.0.8 software at an effect size level of 0.40, α error probability level of 0.05, and a power level of 0.80. Shapiro-Wilk and Levene’s tests were done for data distribution and homogeneity of variance, respectively. Logarithmic transformation was used for data that violated the normal distribution assumptions and homogeneity of variance. Two-way repeated measures ANOVA was first conducted to determine whether intervention (CNO) was significant. When the intervention and/or intervention-by-time interactions were significant, general linear model (GLM) procedures were conducted for post hoc multiple comparisons. Body weight and/or food intake were used as covariates when appropriate^57^. Unpaired t-test or Mann-Whitney U test was done to compare insulin levels and the area under (or above) the curves for GTT/ITT. P ≤ 0.05 was considered significant for the intervention, interaction, and all multiple comparisons. Greenhouse-Geisser correction was used for intervention-by-time interaction if the assumption of sphericity had been violated. The results are presented as mean ± s.e.m.

## Supplementary information

Supplementary information (Uner et al)

## Data Availability

The data supporting the current study are available from the corresponding author Y.B.K. upon request.

## References

[1] Zimmet P, Alberti KG, Shaw J (2001). Global and societal implications of the diabetes epidemic. Nature.

[2] Zimmet P (2000). Globalization, coca-colonization and the chronic disease epidemic: can the Doomsday scenario be averted?. Journal of internal medicine.

[3] Goncalves GH, Li W, Garcia AV, Figueiredo MS, Bjorbaek C (2014). Hypothalamic agouti-related peptide neurons and the central melanocortin system are crucial mediators of leptin’s antidiabetic actions. Cell reports.

[4] Huo L (2009). Leptin-dependent control of glucose balance and locomotor activity by POMC neurons. Cell metabolism.

[5] Shimomura I, Hammer RE, Ikemoto S, Brown MS, Goldstein JL (1999). Leptin reverses insulin resistance and diabetes mellitus in mice with congenital lipodystrophy. Nature.

[6] Uner A (2015). The role of GluN2A and GluN2B NMDA receptor subunits in AgRP and POMC neurons on body weight and glucose homeostasis. Molecular metabolism.

[7] Aponte Y, Atasoy D, Sternson SM (2011). AGRP neurons are sufficient to orchestrate feeding behavior rapidly and without training. Nature neuroscience.

[8] Koch M (2015). Hypothalamic POMC neurons promote cannabinoid-induced feeding. Nature.

[9] Krashes MJ (2011). Rapid, reversible activation of AgRP neurons drives feeding behavior in mice. The Journal of clinical investigation.

[10] Steculorum SM (2016). AgRP Neurons Control Systemic Insulin Sensitivity via Myostatin Expression in Brown Adipose. Tissue. Cell.

[11] Armbruster BN, Li X, Pausch MH, Herlitze S, Roth BL (2007). Evolving the lock to fit the key to create a family of G protein-coupled receptors potently activated by an inert ligand. Proceedings of the National Academy of Sciences of the United States of America.

[12] Urban DJ, Roth BL (2015). DREADDs (designer receptors exclusively activated by designer drugs): chemogenetic tools with therapeutic utility. Annual review of pharmacology and toxicology.

[13] Baver SB (2014). Leptin modulates the intrinsic excitability of AgRP/NPY neurons in the arcuate nucleus of the hypothalamus. The Journal of neuroscience: the official journal of the Society for Neuroscience.

[14] Takahashi KA, Cone RD (2005). Fasting induces a large, leptin-dependent increase in the intrinsic action potential frequency of orexigenic arcuate nucleus neuropeptide Y/Agouti-related protein neurons. Endocrinology.

[15] Cowley MA (2001). Leptin activates anorexigenic POMC neurons through a neural network in the arcuate nucleus. Nature.

[16] Fujikawa T, Chuang JC, Sakata I, Ramadori G, Coppari R (2010). Leptin therapy improves insulin-deficient type 1 diabetes by CNS-dependent mechanisms in mice. Proceedings of the National Academy of Sciences of the United States of America.

[17] Oral EA (2012). Leptin for type 1 diabetes: coming onto stage to be (or not?). Pediatric diabetes.

[18] Xu J (2018). Genetic identification of leptin neural circuits in energy and glucose homeostases. Nature.

[19] Qiu J, Fang Y, Ronnekleiv OK, Kelly MJ (2010). Leptin excites proopiomelanocortin neurons via activation of TRPC channels. The Journal of neuroscience: the official journal of the Society for Neuroscience.

[20] Bouret SG, Draper SJ, Simerly RB (2004). Trophic action of leptin on hypothalamic neurons that regulate feeding. Science.

[21] O’Malley D (2007). Leptin promotes rapid dynamic changes in hippocampal dendritic morphology. Molecular and cellular neurosciences.

[22] Lee SJ (2013). Leptin stimulates neuropeptide Y and cocaine amphetamine-regulated transcript coexpressing neuronal activity in the dorsomedial hypothalamus in diet-induced obese mice. The Journal of neuroscience: the official journal of the Society for Neuroscience.

[23] Coppari R, Bjorbaek C (2012). Leptin revisited: its mechanism of action and potential for treating diabetes. Nature reviews. Drug discovery.

[24] Dhar M (2014). Leptin-induced spine formation requires TrpC channels and the CaM kinase cascade in the hippocampus. The Journal of neuroscience: the official journal of the Society for Neuroscience.

[25] Dhar M (2014). Leptin induces hippocampal synaptogenesis via CREB-regulated microRNA-132 suppression of p250GAP. Molecular endocrinology.

[26] Gavello D, Carbone E, Carabelli V (2016). Leptin-mediated ion channel regulation: PI3K pathways, physiological role, and therapeutic potential. Channels.

[27] Birnbaumer L, Abramowitz J, Brown AM (1990). Receptor-effector coupling by G proteins. Biochimica et biophysica acta.

[28] Zhu X, Birnbaumer L (1996). G protein subunits and the stimulation of phospholipase C by Gs-and Gi-coupled receptors: Lack of receptor selectivity of Galpha(16) and evidence for a synergic interaction between Gbeta gamma and the alpha subunit of a receptor activated G protein. Proceedings of the National Academy of Sciences of the United States of America.

[29] Liu T (2012). Fasting activation of AgRP neurons requires NMDA receptors and involves spinogenesis and increased excitatory tone. Neuron.

[30] Sahu A (2003). Leptin signaling in the hypothalamus: emphasis on energy homeostasis and leptin resistance. Frontiers in neuroendocrinology.

[31] Welters A (2017). NMDAR antagonists for the treatment of diabetes mellitus-Current status and future directions. Diabetes, obesity & metabolism.

[32] Roth BL (2016). DREADDs for Neuroscientists. Neuron.

[33] Donato J, Frazao R, Elias CF (2010). The PI3K signaling pathway mediates the biological effects of leptin. Arquivos brasileiros de endocrinologia e metabologia.

[34] Huang H (2012). Rho-kinase regulates energy balance by targeting hypothalamic leptin receptor signaling. Nature neuroscience.

[35] Huang H, Lee DH, Zabolotny JM, Kim YB (2013). Metabolic actions of Rho-kinase in periphery and brain. Trends in endocrinology and metabolism: TEM.

[36] Morton GJ (2005). Leptin regulates insulin sensitivity via phosphatidylinositol-3-OH kinase signaling in mediobasal hypothalamic neurons. Cell metabolism.

[37] Roman EA (2010). Central leptin action improves skeletal muscle AKT, AMPK, and PGC1 alpha activation by hypothalamic PI3K-dependent mechanism. Molecular and cellular endocrinology.

[38] Williams KW (2011). The acute effects of leptin require PI3K signaling in the hypothalamic ventral premammillary nucleus. The Journal of neuroscience: the official journal of the Society for Neuroscience..

[39] Berglund ED (2012). Direct leptin action on POMC neurons regulates glucose homeostasis and hepatic insulin sensitivity in mice. The Journal of clinical investigation.

[40] Lam DD (2015). Conditional expression of Pomc in the Lepr-positive subpopulation of POMC neurons is sufficient for normal energy homeostasis and metabolism. Endocrinology.

[41] Bumaschny VF (2012). Obesity-programmed mice are rescued by early genetic intervention. The Journal of clinical investigation.

[42] Fenno LE (2014). Targeting cells with single vectors using multiple-feature Boolean logic. Nature methods.

[43] Hill JW (2008). Acute effects of leptin require PI3K signaling in hypothalamic proopiomelanocortin neurons in mice. The Journal of clinical investigation.

[44] Zhan C (2013). Acute and long-term suppression of feeding behavior by POMC neurons in the brainstem and hypothalamus, respectively. The Journal of neuroscience: the official journal of the Society for Neuroscience.

[45] Buettner R, Scholmerich J, Bollheimer LC (2007). High-fat diets: modeling the metabolic disorders of human obesity in rodents. Obesity.

[46] El-Haschimi K, Pierroz DD, Hileman SM, Bjorbaek C, Flier JS (2000). Two defects contribute to hypothalamic leptin resistance in mice with diet-induced obesity. The Journal of clinical investigation.

[47] Houseknecht KL, Baile CA, Matteri RL, Spurlock ME (1998). The biology of leptin: a review. Journal of animal science.

[48] Tschop M, Heiman ML (2001). Rodent obesity models: an overview. Experimental and clinical endocrinology & diabetes: official journal, German Society of Endocrinology [and] German Diabetes Association.

[49] West DB, Boozer CN, Moody DL, Atkinson RL (1992). Dietary obesity in nine inbred mouse strains. The American journal of physiology.

[50] Balthasar N (2004). Leptin receptor signaling in POMC neurons is required for normal body weight homeostasis. Neuron.

[51] Tong Q, Ye CP, Jones JE, Elmquist JK, Lowell BB (2008). Synaptic release of GABA by AgRP neurons is required for normal regulation of energy balance. Nature neuroscience.

[52] van de Wall E (2008). Collective and individual functions of leptin receptor modulated neurons controlling metabolism and ingestion. Endocrinology.

[53] Chung WK, Chua SC, Lee GH, Leibel RL (1997). Polymerase chain reaction-restriction fragment length polymorphisms (PCR-RFLP) and electrophoretic assays for the mouse obese (Lepob) mutation. Obesity research.

[54] Garfield AS (2015). A neural basis for melanocortin-4 receptor-regulated appetite. Nature neuroscience.

[55] Munzberg H, Huo L, Nillni EA, Hollenberg AN, Bjorbaek C (2003). Role of signal transducer and activator of transcription 3 in regulation of hypothalamic proopiomelanocortin gene expression by leptin. Endocrinology.

[56] Ellacott KL, Morton GJ, Woods SC, Tso P, Schwartz MW (2010). Assessment of feeding behavior in laboratory mice. Cell metabolism.

[57] Tschop MH (2011). A guide to analysis of mouse energy metabolism. Nature methods.

