# Supplementary material for: Role of POMC and AgRP neuronal activities on glycaemia in mice
Source: Sci Rep. 2019 Sep 10;9:13068. doi: 10.1038/s41598-019-49295-7 (PMC6736943; doi:10.1038/s41598-019-49295-7)
Supplement: Supplementary file 1 — Supplementary information (Uner et al) [file 41598_2019_49295_MOESM1_ESM.pdf]

<u>SUPPLEMENTARY INFORMATION</u>

Role of POMC and AgRP neuronal activities on glycaemia in mice

Aykut Göktürk Üner, Onur Keçik, Paula G.F. Quaresma, Thiago M. De Araujo, Hyon Lee, Wenjing Li, Hyun Jeong Kim, Michelle Chung, Christian Bjørbæk, and Young-Bum Kim

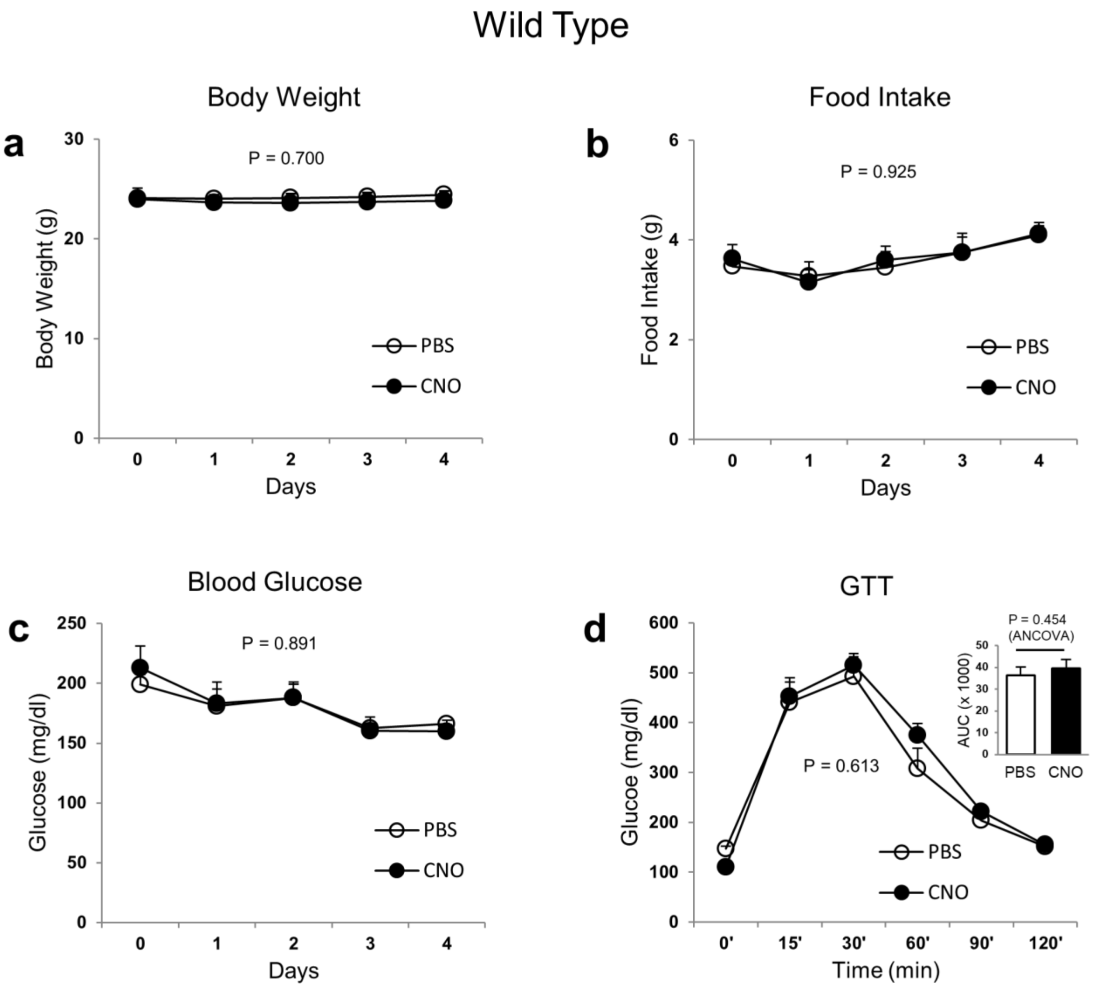

Supplementary figure 1: CNO *per se* does not affect metabolic parameters in wild-type mice. Body weight (A), food intake (B), blood glucose levels (C), and GTT (D) of wild-type (C57BL/6J) mice. Data are shown as means ± s.e.m. (n = 4/group). Repeated measures 2-way ANOVA was done to determine intervention effect. AUC was evaluated with 1-way ANCOVA (body weight was used as covariate). CNO (1 mg/kg, IP) was injected every 8 hours for 5 days. CNO: Clozapine N-oxide. GTT: Glucose tolerance test. AUC: Area under the curve. P values are expressed the intervention (CNO) effect.

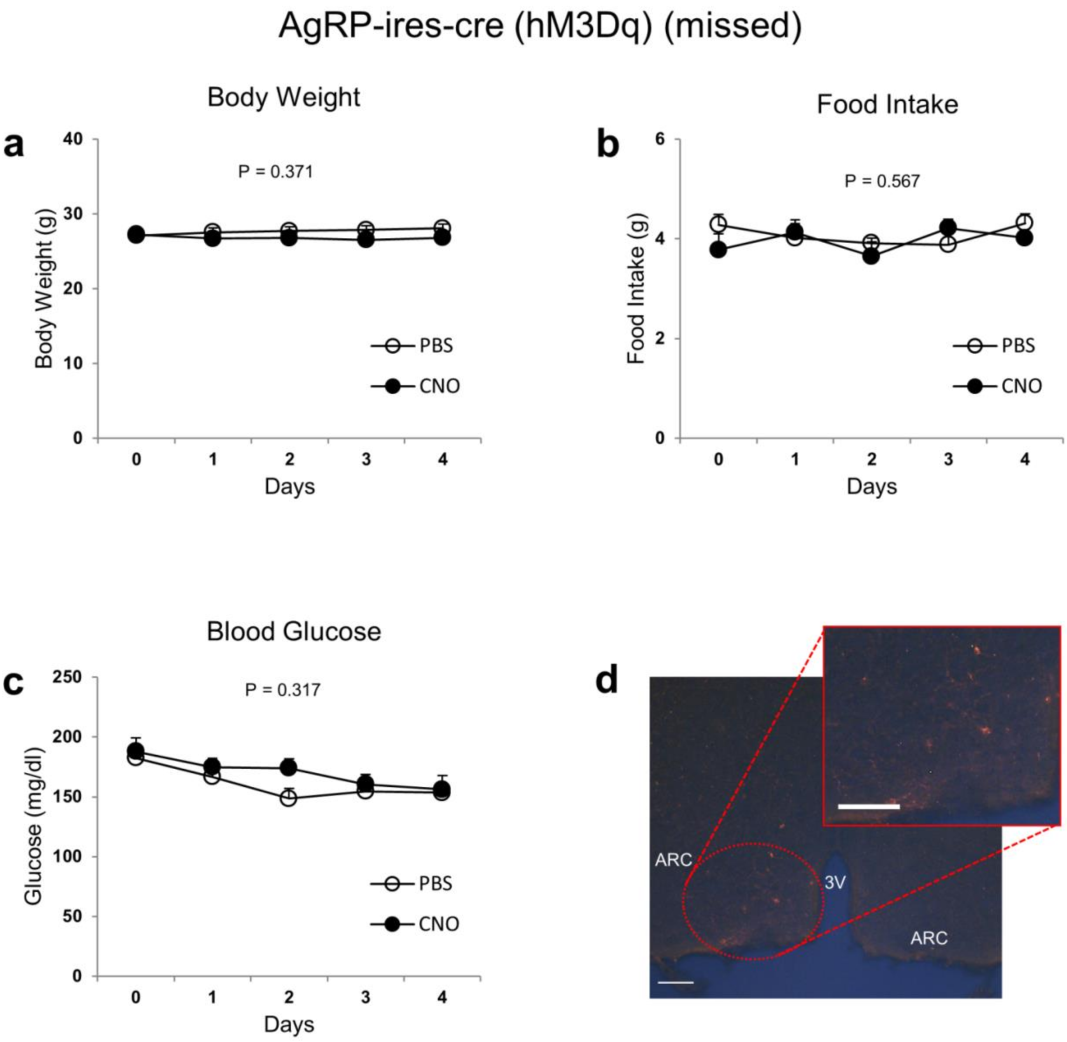

Supplementary figure 2: CNO does not affect metabolic parameters of *AgRP-ires-cre* mice with missed injection AAV8-DIO-hM3Dq-mCherry. Body weight (A), food intake (B), and blood glucose levels (C) of *AgRP-ires-cre* mice. Data are shown as means ± s.e.m. (n = 5-9/group). Image in (D) shows mCherry immunofluorescence. Only a limited number of cells express mCherry. Repeated measures 2-way ANOVA was done to determine intervention effect. CNO (1 mg/kg, IP) was injected every 8 hours for 5 days. CNO: Clozapine N-oxide. ARC: Arcuate nucleus. 3V: Third ventricle. P values are expressed the intervention (CNO) effect. Scale bars, 100 μm.

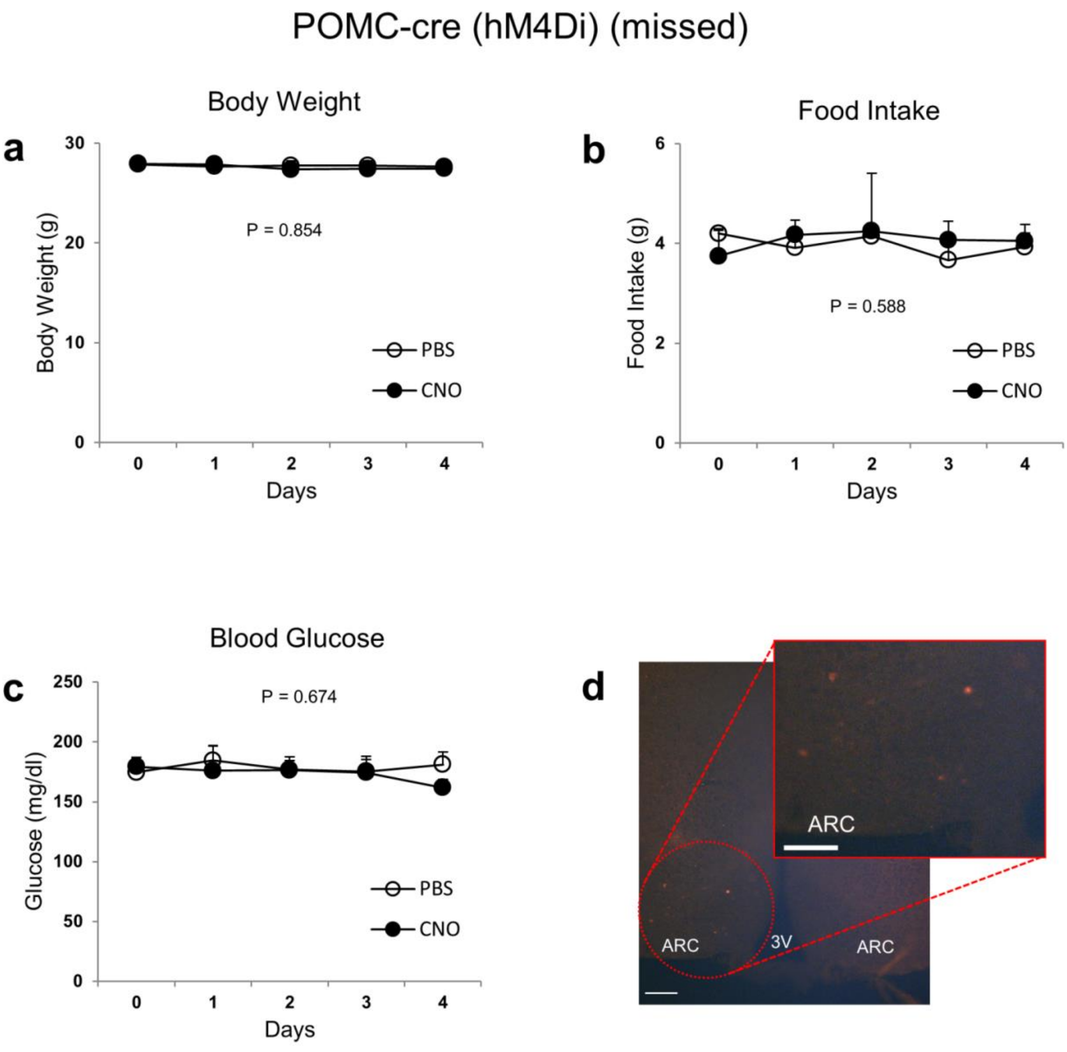

Supplementary figure 3: CNO does not affect metabolic parameters of *POMC-cre* mice with missed injection of AAV8-DIO-hM4Di-mCherry. Body weight (A), food intake (B), and blood glucose levels (C) of *POMC-cre* mice. Data are shown as means ± s.e.m. (n = 5-9/group). Image in (D) shows mCherry immunofluorescence. Only a limited number of cells express mCherry. Repeated measures 2-way ANOVA was done to determine intervention effect. CNO (2.5 mg/kg, IP) was injected every 6 hours for 5 days. CNO: Clozapine N-oxide. ARC: Arcuate nucleus. 3V: Third ventricle. P values are expressed the intervention (CNO) effect. Scale bars, 100 μm.

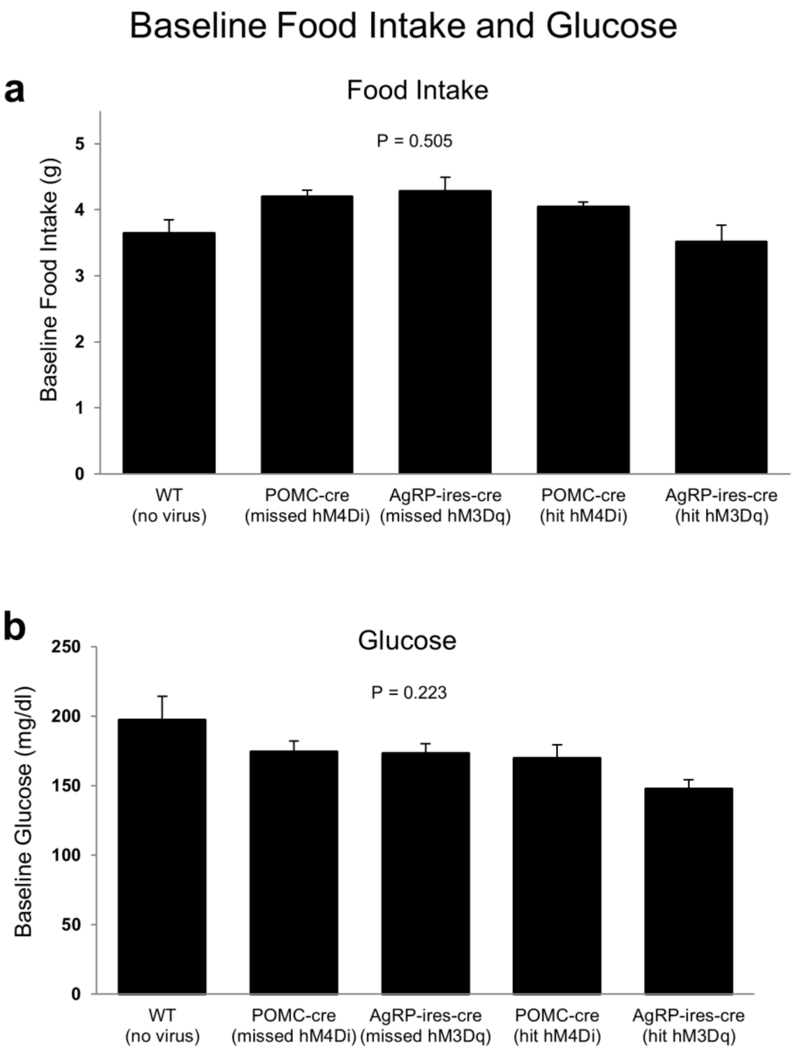

Supplementary figure 4: Food intake and blood glucose levels are comparable between control mice with non-injected or missed injection (missed) and mice correctly injected with hM3Dq or hM4Di viruses (hit). (A and B) Food intake (A) and glucose levels (B) of wild-type (WT), *AgRP-ires-cre* and *POMC-cre* mice injected with activator (AAV8-DIO-hM3Dq-mCherry) or inhibitory (AAV8-DIO-hM4Di-mCherry) virus (hit) or mice injected the viruses, but where the injection site was missed (missed). None of the mice received CNO. Measurements were done two weeks after stereotaxic surgery. Data are shown as means ± s.e.m. (n = 4-9/group). P values were obtained from ANCOVA. Body weights were used as covariate. WT: Wild-type.

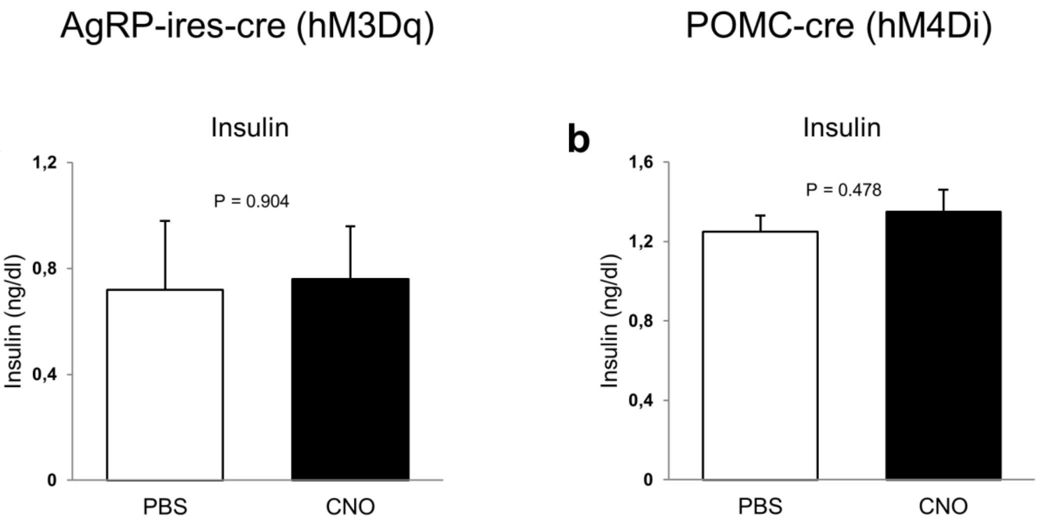

Supplementary figure 5: Activation or inhibition of AgRP or POMC neurons, respectively, does not influence blood insulin levels.
(A and B) Blood insulin levels of *AgRP-ires-cre* mice (A) and *POMC-cre* mice (B). Data are shown as means ± s.e.m. (n = 4-7/group). AAV8-DIO-hM3Dq-mCherry or AAV8-DIO-hM4Di-mCherry was injected into *AgRP-ires-cre* or *POMC-cre* mice, respectively. Serum insulin levels were determined at 1 h after CNO (up to 2.5 mg/kg, IP) and PBS injections. T-test was done for comparing CNO- and PBS-injected mice. CNO: Clozapine N-oxide.

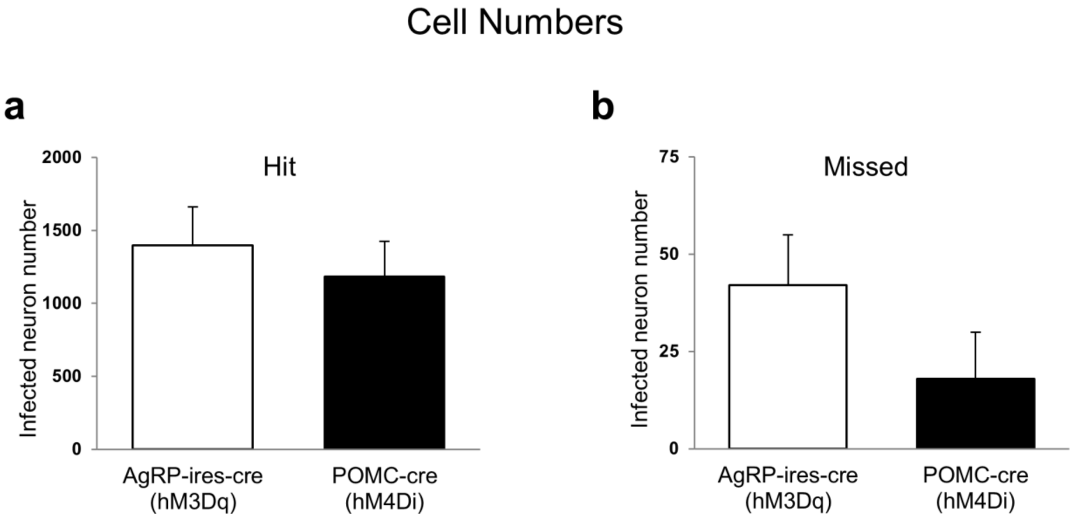

Supplementary figure 6: mCherry positive (infected) cell numbers in AgRP and POMC neurons. Cell counts of mCherry positive AgRP and POMC neurons in the brain sections of *AgRP-ires-cre* and *POMC-cre* mice injected with DREADD virus (AAV8-DIO-hM3Dq-mCherry or AAV8-DIO-hM4Di-mCherry), but where the injection site was hit (A) or missed (B). Data are shown as means ± s.e.m. (n = 5-7/group).
